# Ketamine decreases cell viability of bone explants and impairs bone healing in rats

**DOI:** 10.1186/s13018-020-1579-x

**Published:** 2020-02-11

**Authors:** Dénes B. Horváthy, Péter Szántó, Bence Marschall, Marcell Bagó, Márton Csery, István Hornyák, Attila Doros, Zsombor Lacza

**Affiliations:** 1 Institute of Clinical Experimental Research, Semmelweis University, Budapest, Hungary; 2 Department of Transplantation and Surgery, Semmelweis University, 23-25., Baross str, Budapest, H-1082 Hungary; 3 National Koranyi Institute for Pulmonology, Budapest, Hungary; 4 University of Physical Education, Budapest, Hungary

**Keywords:** Ketamine, Toxicity, Bone, Bone regeneration, Size-specific dose estimation

## Abstract

**Background:**

Ketamine is a widely used anesthetic in experimental medicine. We have also used ketamine for surgical interventions and imaging in rats and found significantly impaired ossification between identically performed experiments, which only differed in the number of anesthetic events. In order to investigate this phenomenon, we estimated the absorbed ionizing radiation and also studied whether ketamine administration has disadvantageous effect on bone cell viability.

**Methods:**

Spongious bone chips and parietal bone disks were harvested from rats. Explants were incubated in stem cell media containing 0.02, 0.2 and 2 mM ketamine. After 3 days of incubation, tetrazolium-based spectrophotometric assay was performed to measure cell viability. Size-specific dose estimation was used to calculate ionizing radiation of computed tomography imaging.

**Results:**

We found that ketamine supplementation with 0.2 mM slightly decreased cell viability, while 2 mM caused significant reduction both in the spongious and cortical explants. The cumulative ionizing radiation was found to be negligible compared to irradiation dosages used to impair ossification.

**Conclusions:**

We conclude that multiple ketamine administration was responsible for the diminished regenerative potential of bone tissue in the present experimental setup. For this reason, we suggest that ketamine anesthesia should be avoided in studies investigating bone regeneration.

## Background

Regeneration is a complex process influenced by uncountable factors, for example, anesthesia-related stress, substances or radiation. The present work is based on an observation from previous experiments investigating bone healing [1, 2]. In order to observe the dynamics of bone remodeling in a rat critical size calvaria model, in vivo computed tomography scans were performed under ketamine–xylazine anesthesia. We observed that bone regeneration in the demineralized bone matrix-filled groups is significantly different between the two identically performed experiments. The only difference was in the number of anesthetic and imaging events. Even though stress itself could effect regeneration, we hypothesized that repeated anesthesia directly influenced the healing capacity of the bone tissue in the present model. In order to investigate this phenomenon, we estimated the absorbed ionizing radiation and also studied whether ketamine administration has disadvantageous effect on the bone cell viability of spongious and cortical explants.

## Methods

### Previous experiments: comparison

Two experimental studies were performed previously with the same surgical and evaluation methods. Briefly, under ketamine–xylazine anesthesia (100–10 mg/kg, Richter Gedeon Plc., Budapest, Hungary; Sigma Aldrich Co., Budapest, Hungary), bone defects were created on the rat calvarium with a terphine bur (4 mm external diameter). Computed tomography (CT) was performed in order to visualize bone healing. In order to perform CT scans, anesthesia was induced again. Demineralized bone matrix-filled defects were compared on the 11th postoperative week, whereas increasing bone density and decreasing remaining bone defect size indicated bone regeneration.

In Experiment 1, until the 11th week, anesthesia was performed 6 times, during surgery and then biweekly [1]. On the 11th week, animals were sacrificed with carbon dioxide, and a last CT scan was performed as well. In Experiment 2, on the other hand, was a long-term investigation, where CT examination was planned only on the 11th and 26th weeks [2]. Therefore, until the 11th postoperative week, anesthesia was induced only once, during surgery.

### Animals

In the present experiment, 230–250 g male Wistar rats (Toxi-Coop co. ltd., Budapest Hungary) were used. The animals were maintained on lab chow and tap water ad libitum in plastic rat cages with 12-h day/night cycle in the animal facility of the Institute of Clinical Experimental Research in Budapest. All procedures performed in studies involving animals were in accordance with the ethical standards of the local Animal Research Committee of Semmelweis University, Budapest, Hungary (Prof. Dr. György Wéber), and the National Scientific Ethical Committee on Animal Experimentation, Budapest, Hungary (Dr. Tamás Brandenburg, date of issue: 2015.02.12., registration number: PEI/001/825–2/2015).

All experimental protocols were approved by the local licensing committee of the Institute of Clinical Experimental Research, Semmelweis University, Budapest, Hungary.

### Spongious and cortical bone explant harvest and culture

Male Wistar rats were euthanized with high concentration of carbon dioxide in a closed plastic chamber. Spongious bone chips of equal size from the femur epicondyle were harvested. Additionally, cortical bone pieces from the parietal bone were cut with a 4-mm internal diameter trephine bur with slow rotational speed and low pressure. Bone pieces were immediately put into pre-heated (37 °C) stem cell culture medium (DMEM supplemented with 1 g/L glutamine, 1 g/L glucose, 10% fetal bovine serum, 100 U/mL penicillin, and 100 μg/mL streptomycin). Bone pieces were separated and cultured under standard cell culture conditions for 3 days (5% CO2, 37 °C). Ketamine (Calypsol, Richter Gedeon Plc., Budapest, Hungary) was added to the medium in 0.02 mM, 0.2 mM, and 2 mM concentrations (*n* = 7/concentration). Ketamine-free medium was used as control (*n* = 14).

### Cell viability: XTT assay

Cellular number was measured with tetrazolium-based assay (Cell proliferation kit II, XTT, Sigma Aldrich Co, Budapest, Hungary). Briefly, after 3 days of culture, media were removed and fresh media (150 μl) containing 50 μl XTT (1:9) were added (37 °C, 1 h). After 4 h of incubation, the absorbance was measured using spectrophotometer at the characteristic wavelength of 492 nm. The background was measured at 650 nm. Absorbance values were normalized with the weight of the bone pieces.

### Radiation dose calculation

Size-specific dose estimation (SSDE) is a known method to calculate absorbed dose of CT scans from the CT dose index (CTDI) and patient size. According to the method provided by the American Association of Physicists in Medicine, a conversion factor can be calculated to estimate radiation dose [3].

$$ \mathrm{SSDE}=\mathrm{conversion}\ \mathrm{factor}\times \mathrm{CTDI} $$

$$ \mathrm{conversion}\ \mathrm{factor}=a\times {e}^{- bx}, $$

$$ \mathrm{where}\ a=1.874799,\kern0.5em b=0.03871313,\kern0.5em x=\mathrm{effective}\ \mathrm{diameter} $$

$$ \mathrm{effective}\ \mathrm{diameter}=\sqrt{\mathrm{anteroposterior}\ \mathrm{diameter}\times \mathrm{laterolateral}\ \mathrm{diameter}} $$

The average rat effective diameter from 15 animals (cm) were calculated. CTDI was 45,7 mGy for every scan. For the present study, CTDI values were given using a 16-cm phantom.

### Statistical analysis

All values are reported as mean ± SEM. One-way ANOVA and Dunnett’s multiple comparison and t-tests were performed using GraphPad Prism statistical software. Probability values of *p* < 0.05 were considered significant.

## Results

### Bone density and remaining bone defect

**Fig. 1:**
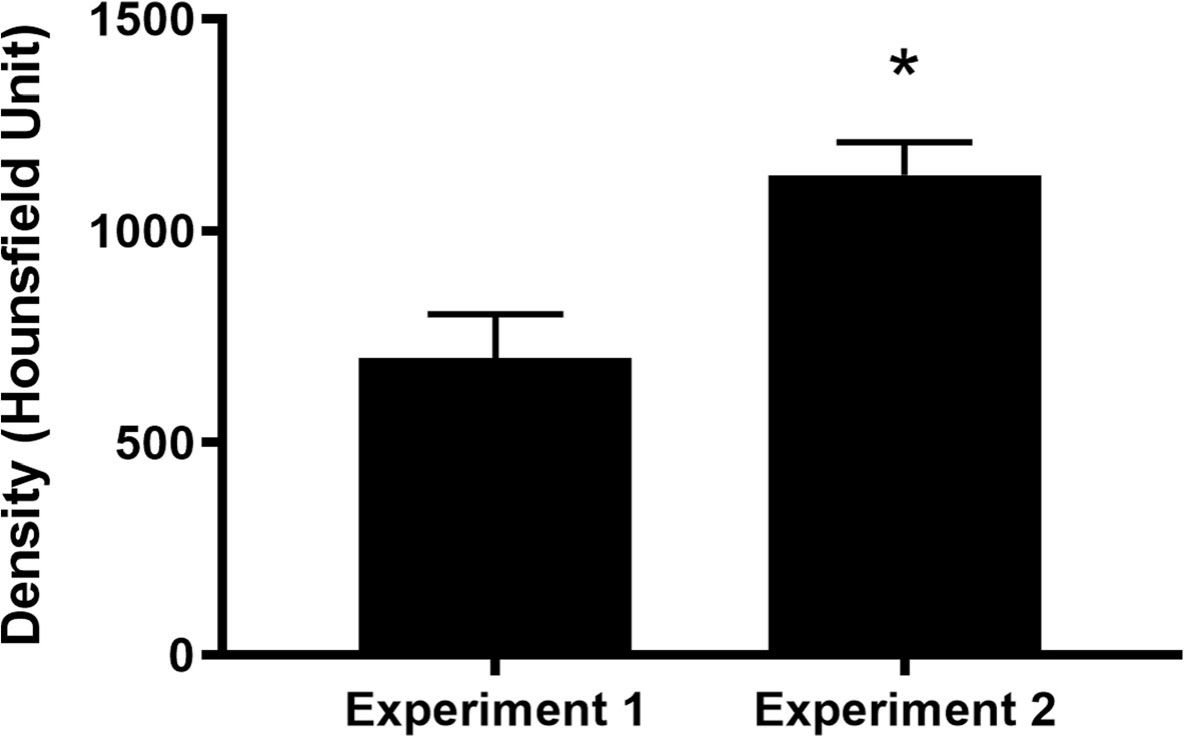
Density of the bone defects after 11 weeks. Significantly lower bone density can be observed on the 11th postoperative week in Experiment 1 with six anesthetic events, which indicates impaired bone healing. The density of the bone defect on the 1st postoperative week is ~ 300 HU. *p < 0.05

Bone density was compared on the 11th postoperative week. In Experiment 1, bone density was 700 ± 105 HU, while those with only one anesthetic event (Experiment 2) showed significantly higher bone density (1132 ± 77 HU, Fig. 1). In addition, in Experiment 1, there was 2.9 ± 1.1 mm^2^ remaining bone defect, while animals in Experiment 2 showed full consolidation without remaining bone defects on the 11th postoperative week (Fig. 1).

### Cell number

**Fig. 2:**
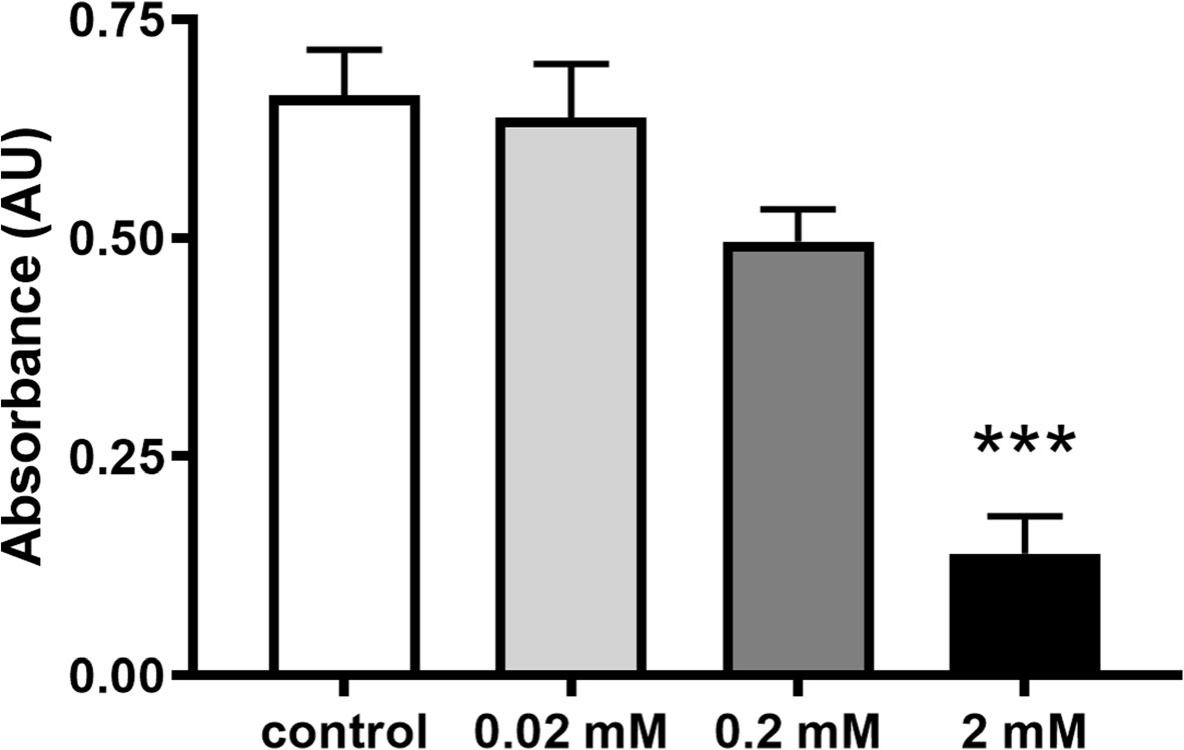
Cell viability of spongious bone explants after ketamine administration. Two millimolars of ketamine significantly decreases cell viability in bone tissue. ***p < 0.001

**Fig. 3:**
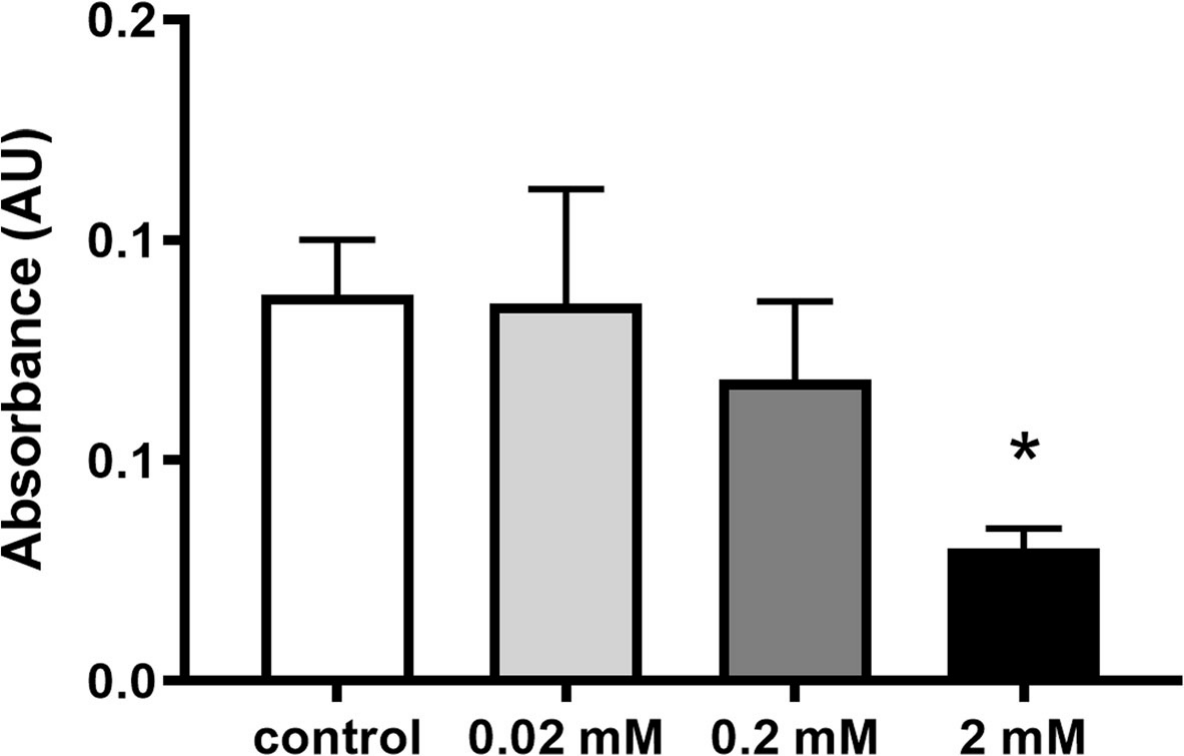
Cell viability of cortical bone explants after ketamine administration. Two millimolars of ketamine significantly decreases cell viability in bone tissue. *: p < 0.05

The mean absorbance of the spongious bone explants in the ketamine-free control group was 0.66 ± 0.05, which did not change after 0.02 mM ketamine supplementation (0.64 ± 0.06). Non-significant reduction in cell viability was noticed after administering 0.2 mM ketamine (0.50 ± 0.04). Adding 2 mM ketamine to the medium, however, significantly decreased cell viability (0.14 ± 0.04) (Fig. 2). Similar tendency was observed with calvaria explants, but due to lower cell density in cortical bone tissue, the absorbance values were smaller (Control: 0.077 ± 0.01, 0.02 mM; 0.07 ± 0.02, 0.2 mM: 0.063 ± 0.01, 2 mM; 0.027 ± 0.003; Fig. 3).

### Radiation dose calculation

The average effective diameter was 3.12 ± 0.2 cm^2^. The conversion factor was 1.66 ± 0.001, meaning, that the SSDE equals to approximately 75.86 mGy for each computed tomography scan in the present experiment. For this reason, the cumulative absorbed dosage for those animals, who have had 5 CT scans was 379.3 mGy.

## Discussion

In the present experiment, we showed that increasing ketamine concentration decreases the number of metabolically active cells on bone explants. This phenomenon could be responsible for the diminished regenerative potential of bone tissue after repeated anesthetic events.

Ionizing radiation could also reduce tissue regeneration. In fact, 20–50 Gy irradiation is generally used to study impaired osteogenesis in rats [4–6]. Lower dose was used by Zhang, who investigated the effects of cosmic radiation on bone morphology in rats [7]. They used 4 × 1 Gy irradiation and found no difference in biomechanical properties and mineral density of rat femora, but showed mild changes in trabecular pattern and bone turnover markers. In the present study, however, a diagnostic protocol was used and the total amount of absorbed dose (SSDE) after 5 CT scans was 379.3 mGy, which is significantly less, than the above-mentioned dosages. For this reason, we concluded that ionizing radiation was not primarily responsible for the reduced osteogenic regeneration in our previous in vivo experiments.

Ketamine on the other hand, was shown to be toxic to hepatocytes, urothelium, and neural cell [8–10]. Additionally, Du reported that clinically relevant concentration of ketamine induces osteoclast apoptosis and inhibits osteoclast formation from bone marrow cultures [11]. Félix showed that ketamine exposure in the developmental phase in Zebra fish results in bone and cartilage malformations [12]. Moreover, Ozturk highlighted that intra-articular ketamine should not be used to control joint pain since it induces apoptosis of chondrocytes in vitro in clinically relevant concentrations [13]. The authors showed that 0.25 mM concentration decreased cell viability, while 0.5 mM, 1 mM, and 2 mM concentrations were definitely toxic to chondrocyte cultures. In the present study, the changes in bone cell viability showed the same tendency with similar concentrations. Namely, non-significant decrease was observed with 0.2 mM, while 2 mM caused significant reduction in cell viability.

## Conclusions

In conclusion, we found decreased bone cell viability after ketamine supplementation in a dose-dependent way in vitro, which suggests that the substance also influences osteogenic progenitor activity in vivo. Other factors like xylazine, radiation, or stress alone could also influence healing, but according to the present results, we conclude that ketamine had a pivotal role in decreasing osteogenic regeneration in our previous in vivo experiments. For this reason, we suggest that ketamine anesthesia should be avoided in studies investigating bone regeneration.

## Data Availability

The datasets generated during and/or analyzed during the current study are available from the corresponding author on reasonable request.

## Abbreviations

CT: Computed tomography
CTDI: CT dose index
Gy: Gray
HU: Hounsfield unit
mGy: Milligray
mM: Millimol/l
SEM: Standard error of mean
SSDE: Size-specific dose estimation
XTT: Sodium 2,3,-bis(2-methoxy-4-nitro-5-sulfophenyl)-5-[(phenylamino)-carbonyl]-2H-tetrazolium
